# Challenges in the diagnosis and management of a ruptured heterotopic gestation following ultrasound-guided embryo transfer in low resource settings: a case report

**DOI:** 10.1186/s13256-023-04317-x

**Published:** 2024-01-24

**Authors:** Ernest Oyeh, Samuel Ofori, Edem K. Hiadzi, Promise E. Sefogah

**Affiliations:** 1 Akai House Clinic, 1 Sixth Circular Rd, Accra, Ghana; 2 Lister Hospital and Fertility Centre, Airport Hills, Accra, Ghana; 3 Department of Obstetrics and Gynaecology, University of Ghana Medical School, Accra, Ghana

**Keywords:** Heterotopic pregnancy, In vitro fertilization and embryo transfer, Assisted reproductive technology, Salpingectomy, Ectopic pregnancy

## Abstract

**Background:**

Heterotopic pregnancies are increasing in incidence with the advent of rising prevalence of in vitro fertilization and embryo transfer (IVF-ET) globally. Although rare, this condition is a serious potentially life-threatening gynaecological complication.

**Case presentation:**

We present the case of a 36-year-old Ghanaian woman who conceived following IVF and presented two weeks after confirmation of intrauterine gestation with sudden onset lower abdominal pain. A diagnosis of ruptured heterotopic pregnancy was made, laparotomy and salpingectomy was done followed with further management of the intrauterine gestation.

**Conclusion:**

To the best of our knowledge, this is the first reported case of heterotopic pregnancy in Ghana. A high index of suspicion for heterotopic pregnancy is required even in the presence of a confirmed intrauterine gestation following IVF-ET.

## Introduction

The simultaneous occurrence of both intrauterine and ectopic pregnancy is termed heterotopic pregnancy [1]. This condition of abnormal implantation can occur following both natural conception and assisted reproductive technique using in vitro fertilization and embryo transfer (IVF-ET). Comparatively, however, the incidence of heterotopic pregnancy remains higher in assisted reproductive technology (ART) cycles (1.5/1000–1/100) than in spontaneous conception (1/10,000–1/50,000) [2, 3]. The incidence of this condition in Ghana is unknown as there has not been any reported case. The fallopian tube still remains the most common site for ectopic gestations (even in ART heterotopic pregnancies), followed by the cornual site as the second most common. Two-thirds of term heterotopic pregnancies result in live birth as do singleton pregnancies but delays in its diagnosis put both mother and the intrauterine gestation at an increased risk [4, 5].

Common risk factors include: previous ectopic gestation with salpingectomy, pelvic inflammatory disease, assisted reproduction, ovarian hyper stimulation, and abortion [6, 7].

Heterotopic pregnancy poses a diagnostic enigma and in low resource settings, this challenge becomes even higher. In practice, the confirmation of an intrauterine gestation excludes ectopic gestation, for which heterotopic pregnancy is rarely considered. This leads to delay in diagnosis until massive haemoperitonium occurs following a rupture of the ectopic gestation. Furthermore, ascites occurring as part of ovarian hyper stimulation in IVF cycles makes it difficult to distinguish haemoperitonium [3, 8]. The clinical presentation of heterotopic pregnancies ranges from mild abdominal pain with nausea and vomiting which occur often in early pregnancy, to haemorrhagic shock with or without adnexal mass [7, 9]. A high index of suspicion is therefore required for prompt diagnosis and appropriate timely intervention to prevent mortality.

Most cases of heterotopic pregnancy are diagnosed and managed in the developed world but the diagnostic challenges and its management difficulties in low resource settings are not very common in literature.

Here, we present a case of heterotopic pregnancy in a nulliparous 36-year-old female who underwent successful IVF treatment cycle, conceived and presented with sudden onset severe lower abdominal pain and vomiting, went into haemorrhagic shock and was successfully managed despite initial diagnostic difficulties.

## Case report

A 36-year old pregnant Ghanaian woman, with Body Mass Index (BMI) = 32 kg/m^2^, gravida 1 para 0 who conceived following an autologous IVF cycle at the Lister Hospital and Fertility Centre in Accra, Ghana, reported at the emergency room with complaints of sudden onset severe lower abdominal pain and an episode of vomiting. The onset of symptoms was 6 h prior to presentation at the emergency room. Her ultrasound-guided (two blastocysts) embryo transfer was done 6 weeks prior to onset of symptoms with a transvaginal ultrasound scan done 2 weeks before presentation, confirming a singleton intrauterine gestational sac. There was no documented features of ovarian hyperstimulation syndrome. Prior to this index pregnancy, she has been trying to conceive for the past 5 years with one unsuccessful intrauterine insemination performed 5 years earlier and another one failed IVF cycle a year earlier. She had vaginal repair for a coital tear 6 years earlier. She also had a history of polycystic ovarian syndrome (PCOS) but no history of smoking, previous history of ectopic gestation, pelvic inflammatory diseases or use of intrauterine devices. Her previous hysterosalpingogram revealed normal uterine cavity with bilateral tubal patency. She had undergone a long gonadotropin release hormone (GnRH) agonist IVF protocol, during which she received 12 days of daily gonadotropin (300iu); human chorionic gonadotropic (hCG) 5000iu trigger and transvaginal ultrasound-guided oocyte retrieval 36 h following the trigger. She had two blastocysts transferred smoothly under ultrasound guidance and continued on progesterone 400 mg twice daily pessaries.

**Fig. 1:**
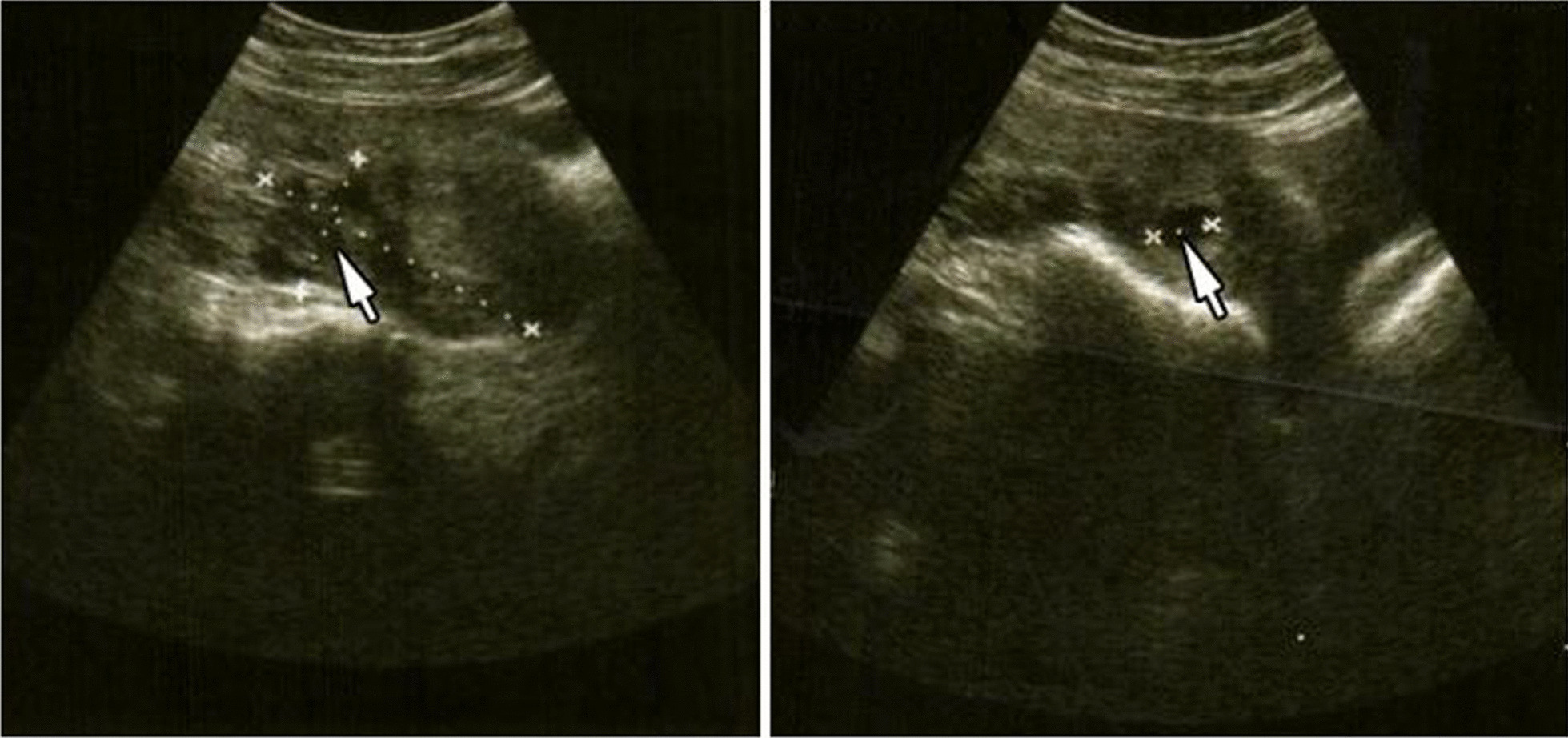
**a** Arrow showing fluid in the right paracolic gutter. **b** Arrow showing an empty intrauterine gestation sac

At the emergency room, she appeared in painful distress, moderately pale, with pulse of 96 beats per minute, and blood pressure of 103/73 mmHg. Her respiratory system findings were unremarkable. The abdomen looked full, with left-sided lower abdominal tenderness, guarding in the left iliac fossa region and normal bowel sounds. An initial clinical suspicion of a possible left ovarian torsion was made. A peripheral venous line was secured and blood samples drawn for a full blood count and serum beta-human chorionic gonadotropin (b-HCG), and intravenous crystalloid infusion commenced. An urgent bedside abdominopelvic ultrasound scan however revealed a slightly bulky uterus with a singleton empty intrauterine gestational sac with a diameter of 15.6 mm (less than 6 weeks gestation) and no foetal pole, about 130 mL of organised haematoma in the pouch of Douglas and about 190 mL of free fluid in the right paracolic gutter. (Fig. 1).

The patient’s condition rapidly worsened within the ensuing 10 min with lethargy, tachycardia (pulse of 108 bpm) and an unrecordable blood pressure. Her full blood count report showed haemoglobin concentration of 7 g/dL, platelet count of 89 × 10^9^/L and a serum beta-HCG of 58,121.31 mIU/L.

Resuscitation was continued with crystalloids and colloids followed by haemotransfusion with 2 units of whole blood, and emergency laparotomy was arranged after informed consent, due to the lack of readily available laparoscopic surgery and its associated very high costs where available in Ghana. The intra-operative findings were: massive haemoperitoneum of approximately about 2 L, ruptured left ampullary gestation with foetus visualised, slightly bulky uterus with normal right tube and ovary. A left salpingectomy was done, haemoperitoneum suctioned, abdomen was lavaged with saline and closed in layers without uterine handling. She was transfused three more units of whole blood post-operatively, administered intramuscular progesterone 100 mg daily, rectal progesterone 400 mg twice daily, intravenous antibiotics and she made a satisfactory recovery. The total duration of surgery was about 1 h 30 min.

**Fig. 2:**
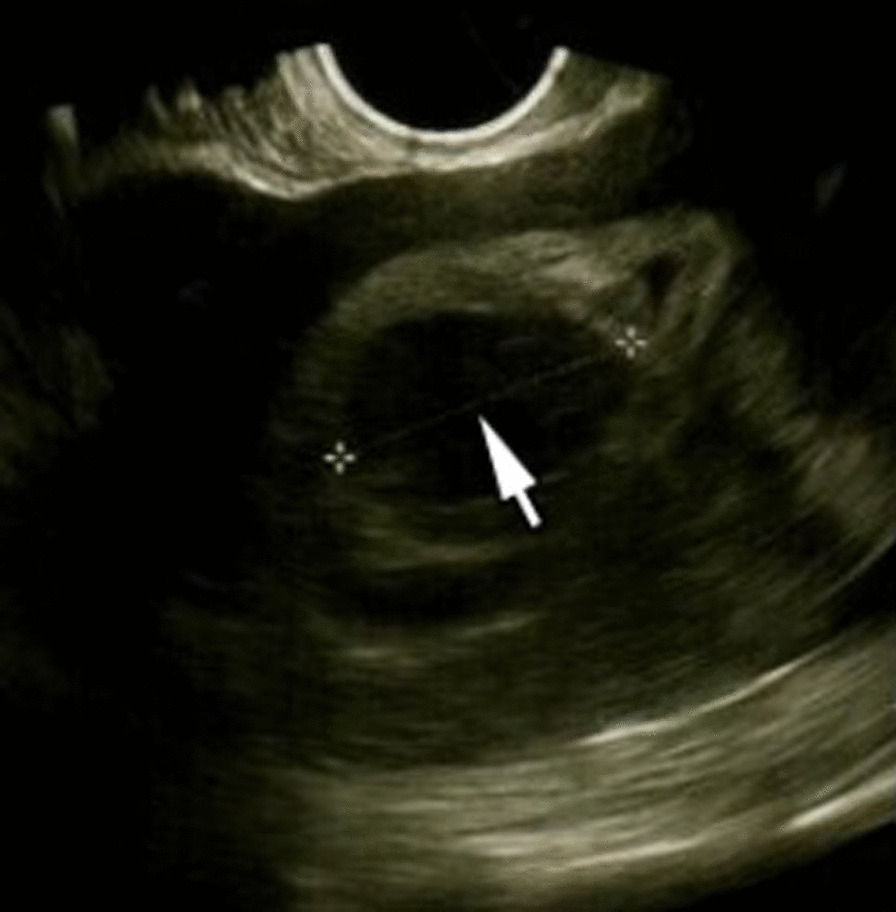
Arrow showing anembryonic gestation 3 days post-laparotomy

On the third day post-surgery, the repeat serum beta-HCG level was 1878.21 mIU/L and a transvaginal ultrasound scan done showed a large irregular gestational sac of diameter 39 mm (8 weeks’ gestation) with absent foetal pole or cardiac activity (Fig. 2). A subsequent diagnosis of an anembryonic intrauterine gestation was made. The patient was counselled appropriately and had a manual vacuum aspiration done to evacuate the retained products of conception. On the post-operative days 4 and 5, the patient experienced repeated episodes of anxiety, palpitation with startling attacks which were effectively managed with the involvement of the Clinical Psychologist. She made a smooth recovery and was discharged home on the fifth day following surgery on oral iron supplementation (Oral Tot’hema 1 vial daily) due to a low haemoglobin level of 8 g/dL and oral antibiotics after completion of 48 h of intravenous antibiotics. Histological examination of specimens confirmed a ruptured tubal pregnancy.

## Discussion

We presented a case of ruptured heterotopic pregnancy following IVF in a patient with prolonged infertility in Ghana.

Heterotopic pregnancies are characterized with significant diagnostic difficulty, in most situations requiring sophisticated diagnostic equipment that may not be readily available in many low-resource settings. The rise in the use of assisted reproductive technology involving controlled ovarian hyperstimulation for the treatment of infertility has led to the apparent increase in incidence of heterotopic pregnancies [9–11]. Following embryo transfer in ART, a routine high resolution transvaginal ultrasound scan is done from 4 to 6 weeks to exclude ectopic or heterotopic pregnancy [12, 13]. In the case presented, this was done and a singleton intrauterine gestation was visualized two weeks prior to her presentation.

Several independent indicators of tubal rupture or active bleeding have been documented, including abdominal pain, rebound tenderness, fluid in pouch of Douglas on transvaginal ultrasound scan (TVS) examination and low blood haemoglobin concentration, [13, 14] among which abdominal pain is the most sensitive predictor [15].

Consistently, our patient presented with an episode of sudden onset abdominal pain and vomiting, associated with tachycardia, hypotension and low blood haemoglobin concentration. Transvaginal ultrasound scan plays a primary role in the diagnosis of heterotopic pregnancy manifesting as coexisting intrauterine gestational sac with adnexal mass or ring sign. Even so, there are some clinical entities that mimic ultrasound scan diagnosis of heterotopic pregnancy posing diagnostic challenges and these include: intrauterine pregnancy with corpus lute cysts, haemorrhagic ovarian cysts, bicornuate uterus gestation, adnexal torsion; surgical conditions such as acute appendicitis and cholecystitis in cyesis [16]. Any of these differential diagnoses can delay diagnosis of heterotopic pregnancy. Abdominopelvic ultrasound scan of the patient in the case reported showed a singleton intrauterine empty gestational sac, organised haematoma in the pouch of Douglas and significant fluid in the right paracolic gutter. This led to serious diagnostic difficulty. Notably, ultrasound scan is not readily available in the emergency room and this require the movement of such patients to the radiology department for establishment of the diagnosis. Also, transvaginal ultrasound is not routinely done due to lack of equipment (transvaginal probes) or the unavailability of sonographers with the technical know-how. Such a situation is very common in low resource settings, [17] and this contributed to the delay of the diagnosis of the patient in this case by additional 6 h from the time of admission. Therefore, eliminating such limitations may have expedited the diagnosis.

The modality of treatment could be medical or surgical depending on the haemodynamic condition of the patient, gestational age and the location of the ectopic gestation. In haemodynamically stable patients, pharmacological interventions under ultrasound guidance can be undertaken, yet, surgical intervention remains the mainstay of management with options such as salpingectomy, salpingotomy, oophorectomy, or hysterectomy on rare occasions. Salpingostomy is the preferred surgical procedure for patients with fertility wishes since salpingectomy normally compromises the fallopian tubes [18]. There is no consensus on the use of laparoscopic or laparotomy as the best method, yet laparoscopy remains the preferred choice [18] with advantages of being minimally invasive. The laparoscopic approach compared to laparotomy method of treating heterotopic pregnancy led to less operation time (40 versus 70 min), less estimated blood loss (150 versus 400 mL) and shortened hospital stay (2 versus 4 days) [19]. However, the prohibition of laparoscopy in hypovolaemic shock is inconclusive [18, 20]. A recent review of ectopic pregnancies in Ghana reported that over 71% were ruptured at the time of presentation, and this happened in this case [21]. We therefore performed laparotomy due to her massive haemoperitoneum, hypovolaemic shock, high cost and the lack of laparoscopic expertise at the time the patient presented to our health facility. A left salpingectomy was done because the tube had ruptured. The patient spent five days on admission before discharged home.

Additionally, the significant haemoperitoneum, time to surgery and having undergone open laparotomy that was prolonged with significant estimated total blood loss requiring blood transfusion necessitated her longer overall hospital stay. Also, there is usually a general lack of access to readily available blood and blood products, making it very difficult to get blood products quickly for transfusion in such low resource settings [22], thus putting the patient at increased risk of morbidity and mortality. In our case presented however, the patient had timely blood products for transfusion from the national blood bank and she made a smooth postoperative recovery.

Either of the surgical methods do not usually interrupt with the intrauterine pregnancy, but there has been a report of 40% loss of the viable intrauterine gestation following the use of surgical methods. In the case presented, the patient was haemodynamically unstable, hence, laparotomy with left salpingectomy was done. Further appropriate management involved surgical evacuation of her retained products of the anembryonic intrauterine gestation 72 h after the laparotomy and salpingectomy [23, 24].

The prompt availability of ultrasound scan enabled the timely confirmation of the diagnosis that made for a timeous surgical intervention to avoid potential mortality.

The patient was subsequently counselled on the risk of recurrence which is considered usually very low, at her next embryo transfer but was nonetheless assured that all necessary precautions would be taken at the embryo transfer as well as reporting any early symptoms following her next conception. For preventing recurrence, the option of prophylactic contralateral salpingectomy has not gained universal consensus. Some have advocated resection of the interstitial portion of the tube to avoid interstitial pregnancy following the prophylactic salpingectomy [25]. This would however expose the patient to uterine rupture in subsequent pregnancy. Therefore, it has been recommended that during the surgery for the first episode, the contralateral tube is inspected and if abnormal the removal done provided the patient had been counselled on same prior to surgery and the patient is stable [5, 25].

## Conclusion

This report describes the conflicting presentation of heterotopic pregnancy which can delay diagnosis. Although rare, heterotopic pregnancy should be considered in the setting of acute abdomen especially in patients who conceived through ART.

## Data Availability

Supporting patient data is available upon request.
